# Global, regional, and national burden of premenstrual syndrome from 1990 to 2021 and projections to 2050: an analysis based on the 2021 Global Burden of Disease study

**DOI:** 10.3389/fpsyt.2025.1644774

**Published:** 2025-12-01

**Authors:** Ruonan Qiang, Linlin Guo, Zheyu Xu, Yuanye Gu, Yanfeng Liu, Yingqiao Wang, Zhinan Liu, Jiayi Liang

**Affiliations:** 1 Dongzhimen Hospital, Beijing University of Chinese Medicine, Beijing, China; 2 Department of Oncology, Longhua Hospital Shanghai University of Traditional Chinese Medicine, Shanghai, China

**Keywords:** premenstrual syndrome, Global Burden of Disease, temporal trend, Socio-demographic Index, prevalence, years lived with disability

## Abstract

**Background:**

Premenstrual syndrome (PMS) imposes significant psychological and mental health burdens on women’s reproductive and general well-being. Disparities in the recognition and reporting of PMS symptoms exist across different regions, influenced by social, cultural, and economic inequalities. This study aims to inform the development of future resource allocation, and ultimately safeguard women's reproductive mental health.

**Methods:**

Utilizing data from the Global Burden of Disease (GBD) 2021 database, we employed Joinpoint regression analysis to examine trends in the burden of PMS from 1990 to 2021, and investigated the impact of the Socio-demographic Index (SDI) on the PMS burden. Additionally, we compared the age distribution characteristics of prevalent PMS cases in 1990 and 2021 and projected the burden to 2050 using the Bayesian Age-Period-Cohort (BAPC) model.

**Results:**

Despite fluctuations, the global burden of PMS was higher in 2021 compared to 1990. The low-middle SDI region consistently had the highest age-standardized prevalence rate (ASPR) and age-standardized years lived with disability (YLDs) rate, which continued to rise; the middle SDI region followed. The high SDI region generally had the lightest burden for most of the period. Among the five SDI regions, only the high-middle SDI region showed a decrease in burden in 2021 compared to 2019. ASPR and age-standardized YLDs rate demonstrated an initial increase followed by a decrease with rising SDI levels. The age distribution of prevalent PMS cases shifted: the peak prevalence moved from the 20–24 age group in 1990 to the 35–39 age group in 2021, while the 40-44 age group was followed by 35-39 in the the same year. Projections showed a declining trend in the global burden of PMS by 2050.

**Conclusion:**

Overall, the global burden of PMS has shown an increasing trend from 1990 to 2021. The burden in low SDI regions may be substantially underestimated, influenced by social, cultural, and economic factors. In socioeconomically disadvantaged regions, attention to menstrual-related mental health, scientific diet, and allocation of healthcare resources require further optimization. In fact, mental health of women aged 35-44 should be emphasized throughout their lifespans, for a better reproductive and general well-being.

## 1 Introduction

Reproductive health is a pivotal determinant of women’s mental health across the lifespan. Menstruation, a key physiological process in women, exhibits cyclical changes closely linked to the risk of adverse psychological and mental states (1). Premenstrual syndrome (PMS) manifests as a constellation of emotional and physical symptoms, including irritability, anxiety, depression, bloating, diarrhea, and breast tenderness. Symptoms characteristically emerge during the luteal phase and resolve within a week after menses onset. When severe emotional lability predominates, it is classified as premenstrual dysphoric disorder (PMDD) (1, 2), formally recognized as a depressive disorder in the Diagnostic and Statistical Manual of Mental Disorders, Fifth Edition (DSM-5) (3). PMS/PMDD imposes significant physical and mental health burdens throughout women's reproductive years, with potential long-term impacts on well-being (4). PMS affects 20-30% of reproductive-aged women globally (5), with up to 90% reporting some premenstrual symptoms; mood swings and anxiety are common, persistent symptoms across age groups (6). Worldwide meta-analyses indicate a PMDD prevalence of 3.2% (7). Although the pathogenesis of PMS remains incompletely elucidated, fluctuations in ovarian hormones are considered central (8).

PMS not only reduces quality of life and social functioning but is also identified as a risk factor for perinatal depression (9). PMDD is associated with a high risk of self-injurious thoughts and behaviors (10). The multifaceted negative impact of PMS on physical, mental, and reproductive health, affecting mood, work efficiency, and interpersonal relationships, translates into considerable economic and health burdens for individuals, families, and society. However, cultural and societal influences often lead women to conceal menstrual-related symptoms (11), particularly emotional ones, creating disparities in symptom expression across regions. Coupled with evolving societal roles and increasing stressors experienced by women globally, this suggests a potentially substantial hidden burden of PMS. Furthermore, significant disparities exist in PMS diagnosis and management across countries and regions with varying levels of socioeconomic development. Therefore, a comprehensive understanding of the PMS burden across different regions and populations is crucial for formulating precise and effective public health policies.

Initiated in 1991, the Global Burden of Disease (GBD) study is a comprehensive global health research project that has provided ongoing, empirically based assessments of health status worldwide for over three decades, with increasing detail in each iteration (12). The widespread COVID-19 pandemic has had long-term, severe impacts on population health globally. GBD 2021 introduced, for the first time, estimates of the health loss attributable to the COVID-19 pandemic. Notably, depressive and anxiety disorders dominated the years lived with disability (YLDs) burden in 2020 and 2021, with a greater burden observed in females than males (13).

Although existing GBD studies report a surge in global prevalent PMS cases and YLDs from 1990 to 2019, while age-standardized prevalence rate (ASPR) and age-standardized YLDs rate remained relatively stable (14), due to data constraints in earlier database versions not yet updated through 2021, previous analyses primarily focused on descriptive trends from 1990 to 2019. The COVID-19 pandemic has exerted substantial adverse effects on women’s mental health. The incorporation of 2020 and 2021 data in the GBD 2021 release now enables a more accurate estimation of the PMS burden in the post-pandemic context. Unlike previous studies on this topic, our research examines PMS burden trends from 1990 through 2021 and extends projections of disease burden development through 2050. In summary, this study leverages GBD data spanning 1990–2021 to conduct a comprehensive analysis, which aims to conduct a comprehensive analysis using GBD 2021 data to: 1) Analyze trends in ASPR, age-standardized incidence rate (ASIR), and age-standardized YLDs rate of PMS among females globally, stratified by region, Socio-demographic Index (SDI), and age; 2) Assess cross-national changes in PMS burden inequality, particularly concerning SDI, during 1990–2021 and investigate the correlation between SDI and PMS burden. 3) this study will project future PMS burden trends up to 2050, thereby aiding a comprehensive assessment of the global PMS burden across future time periods and anticipating its developmental trajectory, aiming to provide epidemiological evidence for global PMS prevention and control, and inform the development of targeted intervention strategies and future resource allocation, and ultimately safeguard women’s reproductive mental health.

## 2 Methods

### 2.1 Data sources

Data for this study were sourced from the GBD 2021 study. GBD 2021 provides estimates of prevalence, incidence, YLDs, mortality, and disability-adjusted life years for 371 diseases and injuries across 204 countries and territories (13). For most diseases and injuries, prevalence and incidence data were modeled using DisMod-MR 2.1, a Bayesian meta-regression tool. YLDs quantify non-fatal health loss and are calculated by multiplying the estimated age-, sex-, location-, and year-specific prevalence counts of each non-fatal sequela (consequence of a disease or injury) by its corresponding disability weight, derived through a microsimulation process. The SDI is a composite measure of lag-distributed income per capita, average years of education among individuals aged 15 or older, and the total fertility rate among females under 25 (13). It serves as a macro-level indicator of national or regional development, calculated as the geometric mean of indices ranging from 0 (lowest) to 1 (theoretically highest) and categorized into five quintiles (15).

We extracted estimates of prevalence, incidence, YLDs, and their corresponding 95% uncertainty intervals (UIs) for PMS from the GBD 2021 database (https://vizhub.healthdata.org/gbd-results/). The GBD 2021 dataset adheres to standardized protocols and employs advanced statistical modeling; no manual data supplementation or calculation was performed in this study. As PMS is a menstruation-related condition, we included data for females aged 10–54 from the GBD 2021 database. PMS is coded as GA34.4 in the Eleventh Revision of the International Classification of Diseases (ICD-11).

### 2.2 Statistical analyses

Joinpoint regression analysis (Joinpoint Regression Program, Version 5.2.0.0, National Cancer Institute, USA) was employed to analyze temporal trends in PMS burden from 1990 to 2021, stratified by the five SDI quintiles. We used the average annual percentage change (AAPC) and its 95% confidence interval (CI) to quantify long-term trends over the entire period (1990–2021). The AAPC is computed as the geometric weighted average of the annual percentage changes (APCs) from the Joinpoint model, providing a single summary measure of the trend (e.g., an AAPC of 0.3 indicates an average annual increase of 0.3%) (16). Statistical significance was set at *P* < 0.05. Spearman correlation analysis was used to assess the correlation between SDI and ASPR/age-standardized YLDs rate, supplemented by locally estimated scatterplot smoothing (LOESS) regression to explore the expected relationship. The Bayesian Age-Period-Cohort (BAPC) model was utilized to assess and project PMS burden from 2022 to 2050. Compared to other projection models, the BAPC model incorporates the Integrated Nested Laplace Approximation (INLA) method (17), accounting for future global population changes, and offers advantages in prediction coverage and accuracy. All statistical analyses were conducted using R software (version 4.4.2). Data visualization was primarily created using the ggplot2 package, while the nordpred, INLA, BAPC and other packages were used for BAPC modeling.

## 3 Results

### 3.1 Disease burden trends for PMS globally and in the 5 SDI regions from 1990 to 2021

**Figure 1:**
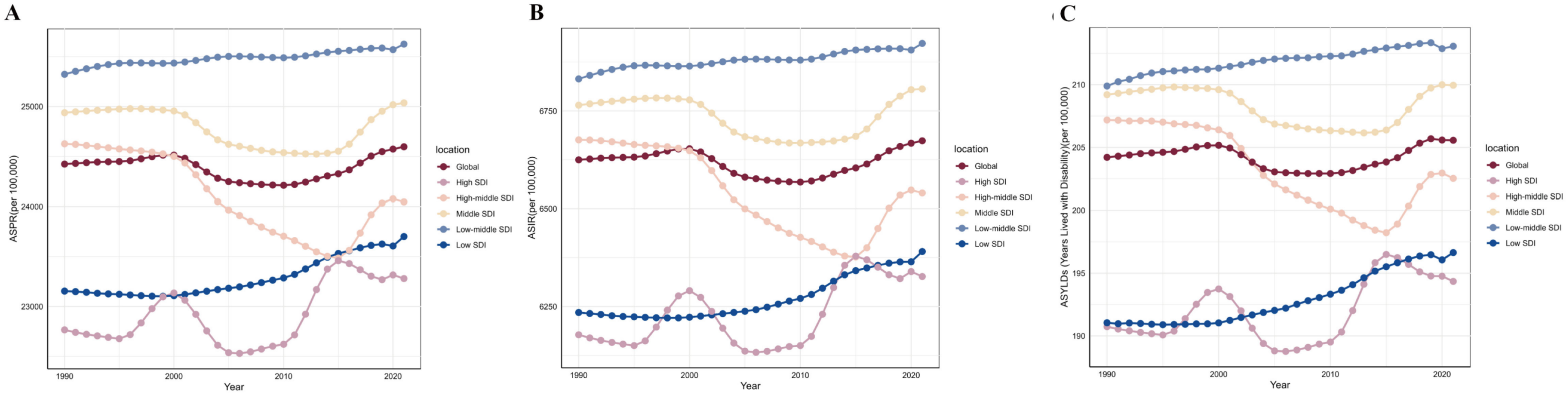
The trends of premenstrual syndrome ASPR, ASIR, and age-standardized YLDs rate among different SDI quintiles. **(A)** ASPR. **(B)** ASIR. **(C)** Age-standardized YLDs rate. ASPR, age-standardized prevalence rate; ASIR, age-standardized incidence rate; YLDs, years lived with disability; SDI, Socio-demographic Index.

Globally, the ASPR of PMS increased from 24,423.22 (95% UI: 20,075.78, 28,720.46) per 100,000 in 1990 to 24,596.70 (95% UI: 20,161.58, 29,027.18) per 100,000 in 2021. The ASIR rose from 485,565.85 (95% UI: 463,307.79, 512,105.95) per 100,000 in 1990 to 488,681.55 (95% UI: 467,179.37, 513,640.54) per 100,000 in 2021. Similarly, the age-standardized YLDs rate showed an increasing trend, from 204.21 (95% UI: 124.38, 311.28) per 100,000 in 1990 to 205.57 (95% UI: 125.21, 313.94) per 100,000 in 2021. Thus, globally, all three metrics increased in 2021 compared to 1990, including a U-shaped trend period (initially decreasing, then increasing). The ASIR showed the largest increase [AAPC: 0.024 (95% UI: 0.021 to 0.026)], while ASPR and age-standardized YLDs rate increased at similar rates [AAPC: 0.02 (0.02 to 0.02)] (**Table 1**; **Figure 1**).

**Table 1:** Disease burden trends for PMS globally and in the 5 SDI regions from 1990 to 2021.

Location	Age-standardized prevalence rate				Age-standardized incidence rate				Age-standardized years lived with disability rate			
	1990 per 100000 (95%UI)	2021 per 100000 (95%UI)	AAPC (95%CI)	*P-*value	1990 per 100000 (95%UI)	2021 per 100000 (95%UI)	AAPC (95%CI)	*P-*value	1990 per 100000 (95%UI)	2021 per 100000 (95%UI)	AAPC (95%CI)	*P-*value
Global	24423.22 (20075.78, 28720.46)	24596.70 (20161.58, 29027.18)	0.02 (0.02 to 0.02)	<0.01	485565.85 (463307.79,512105.95)	488681.55 (467179.37,513640.54)	0.024 (0.021 to 0.026)	<0.01	204.21 (124.38, 311.28)	205.57 (125.21, 313.94)	0.02 (0.02 to 0.02)	<0.01
High-middle SDI	24626.28 (20198.75, 29098.27)	24046.32 (19586.61, 28630.16)	-0.08 (-0.08 to -0.07)	<0.01	409720.94 (389031.26,434663.99)	412517.61 (391907.00,436666.01)	-0.065 (-0.070 to -0.060)	<0.01	207.18 (126.46, 316.74)	202.53 (123.40, 311.07)	-0.07 (-0.08 to -0.07)	<0.01
High SDI	22765.65 (18465.77, 27244.80)	23280.02 (18885.58, 27876.04)	0.07 (0.06 to 0.08)	<0.01	482973.52 (454482.35,515920.81)	480251.64 (451770.03,511002.81)	0.075 (0.065 to 0.085)	<0.01	190.73 (115.83, 288.79)	194.34 (118.23, 294.87)	0.06 (0.05 to 0.07)	<0.01
Low-middle SDI	25322.93 (21079.66, 29320.82)	25625.29 (21169.33, 30046.30)	0.04 (0.03 to 0.04)	<0.01	549065.25 (526198.16,575948.42)	536118.75 (515567.64,560192.41)	0.038 (0.035 to 0.041)	<0.01	209.89 (127.83, 319.23)	213.07 (129.76, 324.79)	0.05 (0.04 to 0.05)	<0.01
Low SDI	23154.27 (19242.73, 26928.49)	23700.80 (19585.93, 27797.08)	0.07 (0.07 to 0.07)	<0.01	591274.94 (568821.51,617657.47)	581690.49 (559923.56,604909.52)	0.076 (0.071 to 0.079)	<0.01	191.05 (116.23, 288.58)	196.64 (120.19, 299.21)	0.09 (0.09 to 0.10)	<0.01
Middle SDI	24937.96 (20561.56, 29268.04)	25036.59 (20563.68, 29525.56)	0.01 (0.01 to 0.02)	<0.01	458263.46 (436239.76,483798.15)	458308.17 (436996.90,482752.13)	0.020 (0.017 to 0.023)	<0.01	209.20 (127.72, 319.99)	209.96 (128.21, 321.38)	0.01 (0.01 to 0.01)	<0.01

Among the five SDI quintiles in 2021, the high, middle, low-middle, and low SDI regions showed increases in ASPR, ASIR, and age-standardized YLDs rate compared to 1990. The low-middle SDI region consistently had the highest burden throughout 1990-2021, with a rising trend; the middle SDI region followed. Despite also showing an increasing trend, the high SDI region generally had the lightest burden for most of the period. Only the high-middle SDI region showed a decrease in burden in 2021 compared to 2019, following a trend of initial decrease and subsequent increase. In 2021, the order of regions from highest to lowest burden for ASPR, ASIR, and age-standardized YLDs rate was: low-middle SDI, middle SDI, global average, high-middle SDI, low SDI, and high SDI (**Table 1**; **Figure 1**).

**Figure 2:**
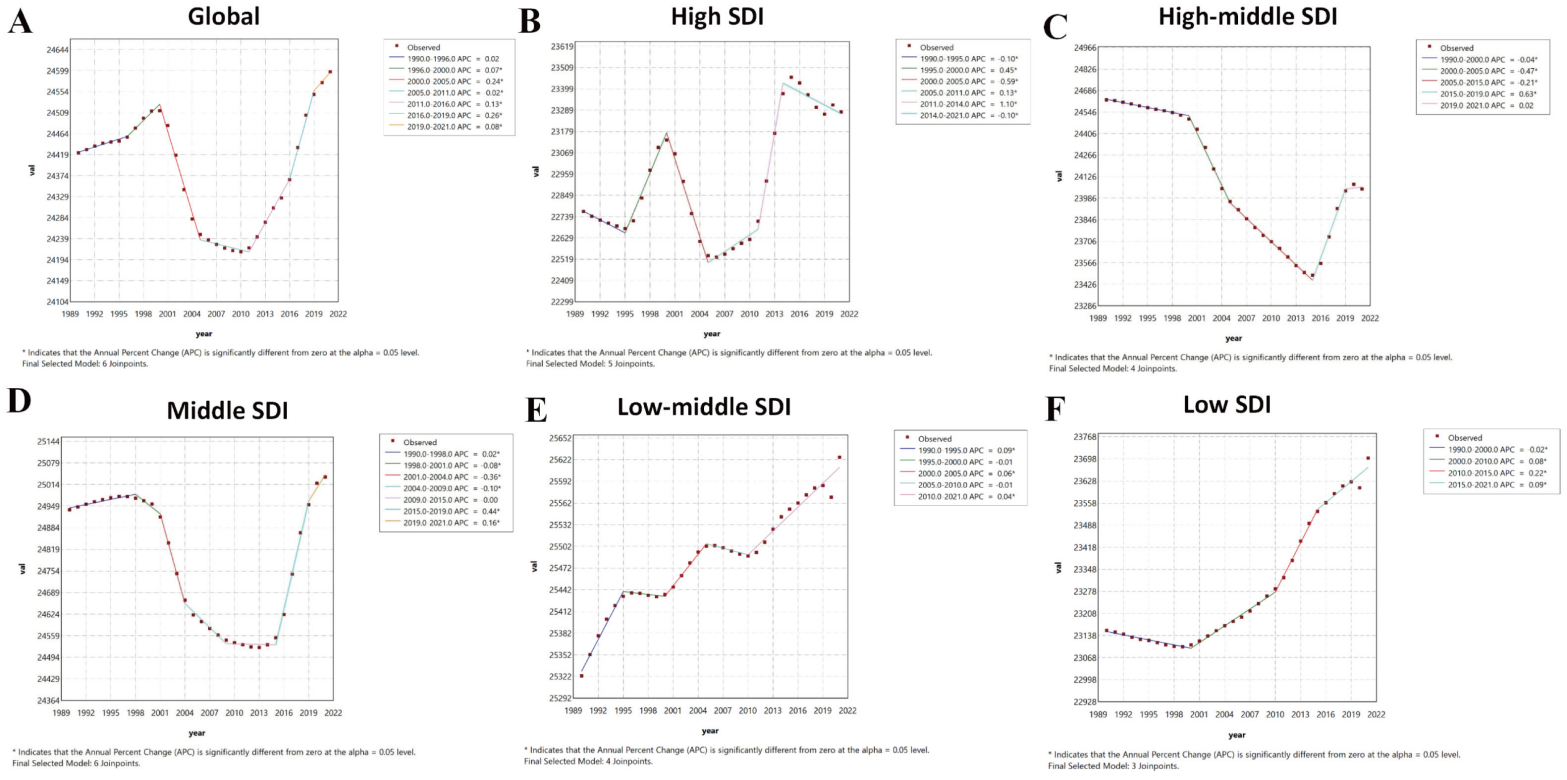
Joinpoint regression analysis results of age-standardized prevalence rate from 1990 to 2021. * represents statistical significance *p* < 0.05. **(A)** Global; **(B)** High SDI; **(C)** High-middle SDI; **(D)** Middle SDI; **(E)** Low-middle SDI; **(F)** Low SDI. SDI, Socio-demographic Index.

Joinpoint regression identified significant inflection points in the burden of PMS based on ASPR. Globally, an increasing trend from 1990–2000 was followed by a sharp decline from 2000-2005 (APC: -0.24), a slower decline from 2005-2011 (APC: -0.02), and a rising trend from 2011 onwards. The high SDI region showed fluctuating trends: decrease (1990-1995; APC: -0.1), sharp increase (1995-2000; APC: 0.45), sharp decrease (2000-2005; APC: -0.59), variable increases (2005–2014), and another decrease (post-2014; APC: -0.1). The high-middle SDI region showed varying degrees of decrease from 1990-2015, followed by an increasing trend after 2015. The middle SDI region increased slowly from 1990-1998 (APC: 0.02), then exhibited a U-shaped trend: decreasing variably from 1998–2009, then remained stable until 2015, and increasing thereafter. The low-middle SDI region increased from 1990-1995 (APC: 0.09), decreased slowly from 1995-2000 (APC: -0.01), increased again from 2000-2005 (APC: 0.06), decreased until 2010, and then increased steadily (APC: 0.04). The low SDI region decreased slowly from 1990-2000 (APC: -0.02) and then showed a fluctuating increase thereafter (**Figure 2**).

**Figure 3:**
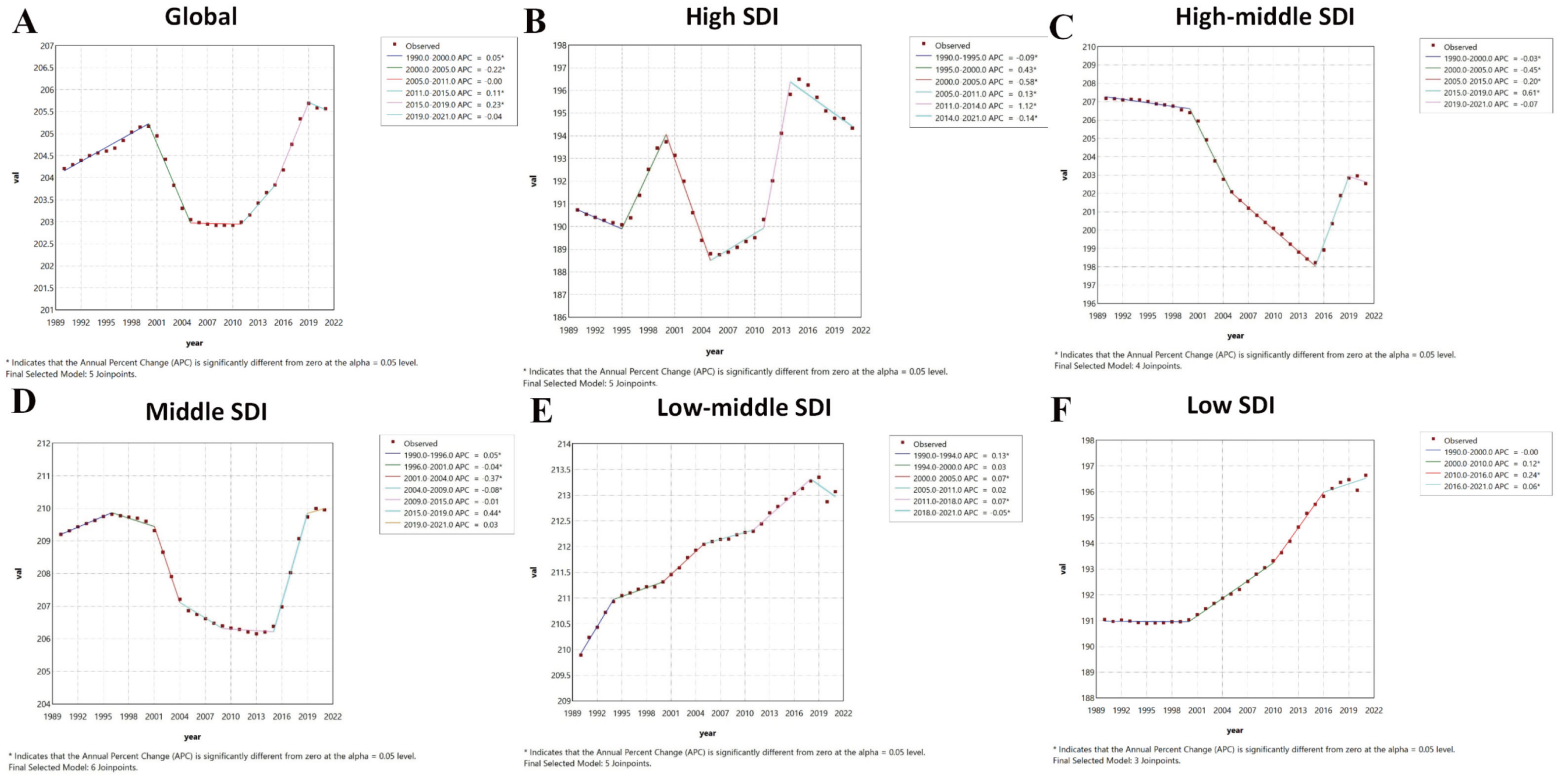
Joinpoint regression analysis results of age-standardized years lived with disability rate from 1990 to 2021. * represents statistical significance p < 0.05. **(A)** Global; **(B)** High SDI; **(C)** High-middle SDI; **(D)** Middle SDI; **(E)** Low-middle SDI; **(F)** Low SDI. SDI, Socio-demographic Index.

Joinpoint analysis for age-standardized YLDs rate revealed trends largely consistent with ASPR globally and across SDI regions, with minor differences in the timing of inflection points. Globally, age-standardized YLDs rate increased slowly from 1990-2000 (APC: 0.05), then transitioned to a decrease (APC: -0.22) until 2005, then remained stable to 2011, and subsequently increased variably in 2011-2019, and decreased in 2019-2021. The high SDI region had inflection points in 1995, 2000, 2005, 2011, and 2014: slow decrease (1990-1995; APC: -0.09), sharp increase (1995-2000; APC: 0.43), rapid decrease (2000-2005; APC: -0.58), variable increases from 2005-2014, and another decrease (post-2014; APC: -0.14). The high-middle SDI region had inflection points in 2000, 2005, 2015, and 2019: predominantly decreasing before 2015, rapid increase (2015-2019; APC: 0.61), and gradual decrease thereafter. The middle SDI region showed a slow increase (1990-1996; APC: 0.05), followed by a U-shaped trend (like ASPR) with inflection points in 2001, 2004, 2009, 2015, and 2019: variable decreases from 1996-2015, rapid increase (2015-2019; APC: 0.44), and slowed increase thereafter. The low-middle SDI region had inflection points in 1994, 2000, 2005, 2011, and 2018: overall increasing trend with phases and variable rates before 2018, followed by a gradual decrease (APC: -0.05). The low SDI region was stable from 1990-2000 and then increased variably (**Figure 3**).

### 3.2 Regional and national burden trends of PMS

**Figure 4:**
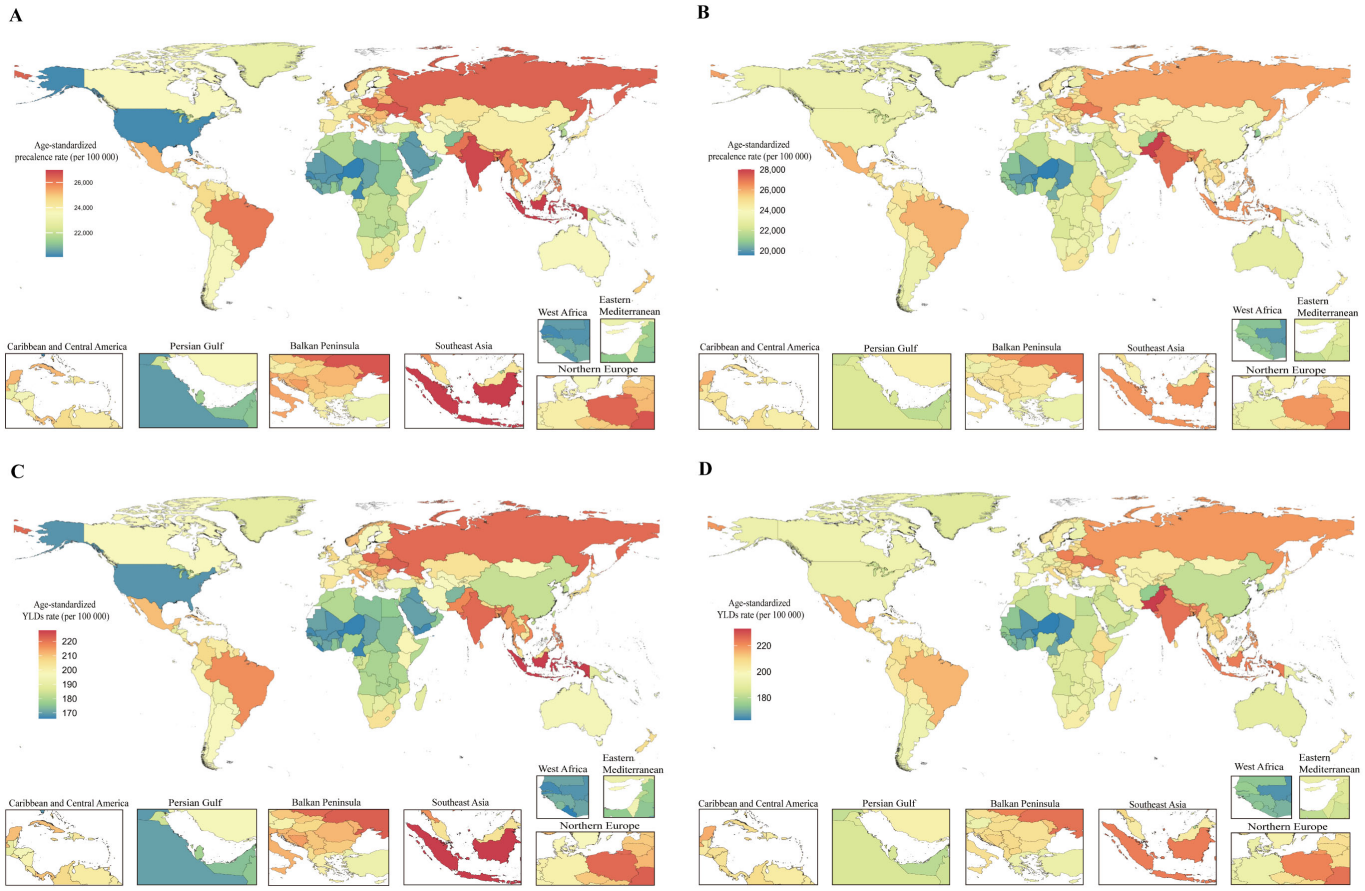
Global burden of disease for PMS in 204 countries and territories based on ASPR and age-standardized YLDs rate for 1990 and 2021. **(A)** 1990 ASPR; **(B)** 2021 ASPR; **(C)** 1990 Age-standardized YLDs rate; **(D)** 2021 Age-standardized YLDs rate. ASPR, age-standardized prevalence rate; YLDs, years lived with disability.

The burden of PMS varied substantially globally in 2021. Among 204 countries and territories, the Islamic Republic of Pakistan had the highest ASPR [28,013.65 (95% UI: 23,707.55, 31,963.41) per 100,000], followed by the Republic of India [27,105.68 (95% UI: 22,576.13, 31,533.57) per 100,000]. The Republic of the Niger had the lowest ASPR [19,560.86 (95% UI: 15,925.97, 23,296.55) per 100,000]. Similarly, the Islamic Republic of Pakistan had the highest age-standardized YLDs rate in 2021 [232.89 (95% UI: 141.64, 355.82) per 100,000], followed by Ukraine [225.11 (95% UI: 136.25, 345.15) per 100,000] and the Republic of India [224.98 (95% UI: 137.47, 343.61) per 100,000]. Consistent with ASPR, the Republic of the Niger had the lowest age-standardized YLDs rate [162.80 (95% UI: 99.21, 249.27) per 100,000] (**Figure 4**; **Supplementary Table S1**).

Regarding the magnitude of change in PMS burden from 1990 to 2021, among 21 GBD regions, High-income North America showed the largest increase in both ASPR [AAPC: 0.41 (0.38 to 0.43)] and age-standardized YLDs rate [AAPC: 0.37 (0.34 to 0.39)]. Western Europe exhibited the largest decrease in both ASPR [AAPC: -0.10 (-0.11 to -0.10)] and age-standardized YLDs rate [AAPC: -0.10 (-0.11 to -0.10)] (**Supplementary Table S2**). At the national level, the top three countries with the largest increases in both ASPR and age-standardized YLDs rate were the United States of America [ASPR AAPC: 0.46 (0.43 to 0.49); age-standardized YLDs rate AAPC: 0.42 (0.39 to 0.45)], followed by the Union of the Comoros [ASPR AAPC: 0.29 (0.28 to 0.30); age-standardized YLDs rate AAPC: 0.30 (0.29 to 0.31)], and the Kingdom of Saudi Arabia [ASPR AAPC: 0.29 (0.28 to 0.29); age-standardized YLDs rate AAPC: 0.29 (0.28 to 0.30)]. Rankings diverged thereafter. Countries and territories with an AAPC of approximately zero for ASPR (though mostly non-significant) included the Republic of Moldova, Republic of Turkey, Hungary, Northern Mariana Islands, Republic of Guinea-Bissau, Republic of the Gambia, Dominican Republic, and Republic of Guinea. For age-standardized YLDs rate, locations with an AAPC of zero included Poland, Barbados, Suriname, North Macedonia, Antigua and Barbuda, Dominican Republic, Republic of Moldova, Northern Mariana Islands, and Republic of Ghana; only Poland’s AAPC was statistically significant. The Republic of Benin and the Republic of Korea showed the largest decreasing trends in ASPR [AAPC: -0.13 (-0.14 to -0.12) and AAPC: -0.13 (-0.19 to -0.08), respectively]. For age-standardized YLDs rate, Greece, Singapore, Republic of Chad, Germany, France, Israel, and the Republic of Korea exhibited the largest decreases (**Figure 4**; **Supplementary Table S1**).

### 3.3 Age pattern of PMS

**Figure 5:**
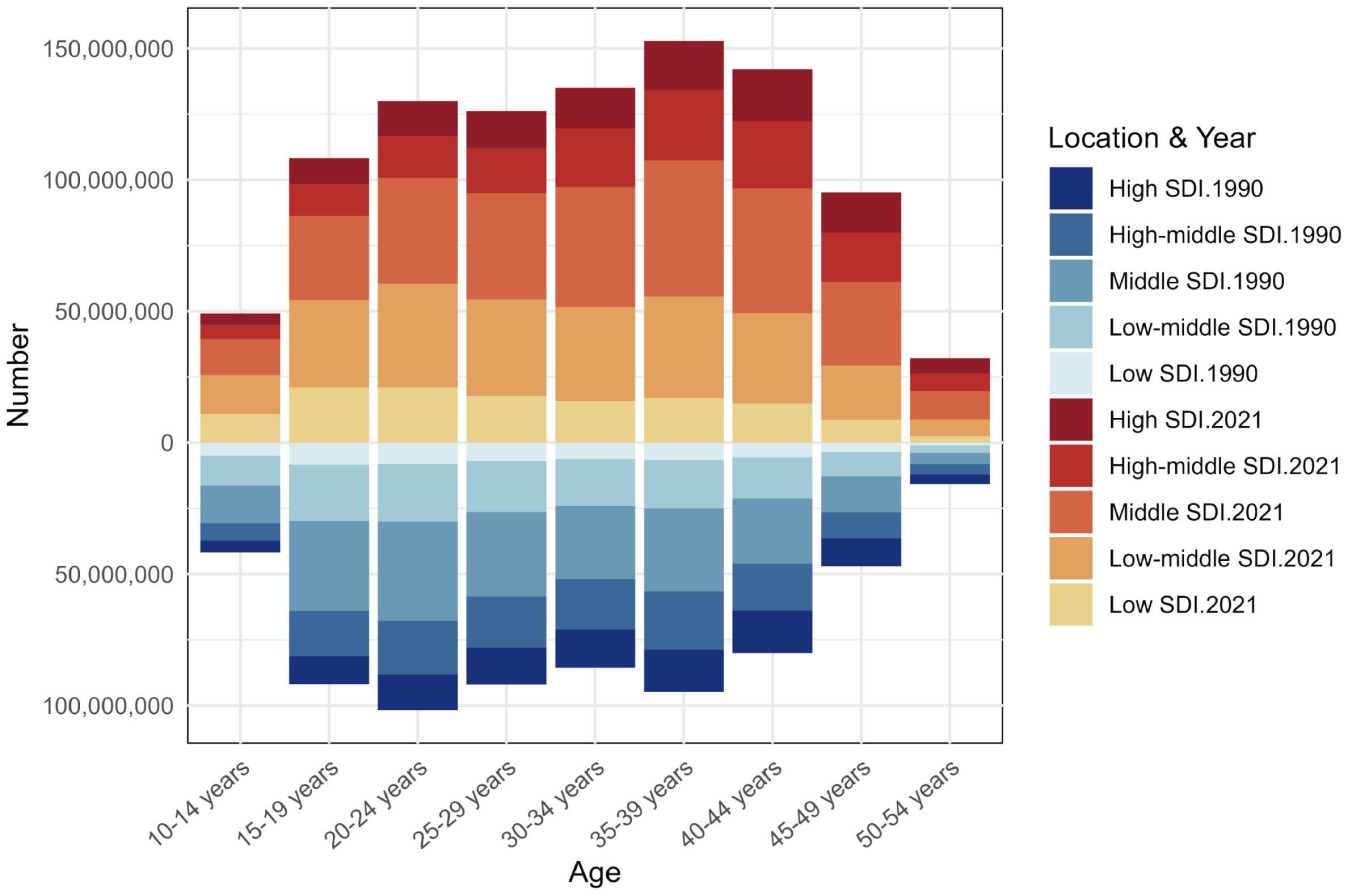
The premenstrual syndrome prevalence number among different age groups in 1990 and 2021.

Analysis of the age distribution of prevalent PMS cases revealed a unimodal pattern in both 1990 and 2021. However, the peak shifted: in 1990, the highest prevalence was in the 20–24 age group, followed by 35-39, 25-29, 15-19, with the lowest in 50–54 years. By 2021, the peak prevalence occurred in the 35–39 age group, followed by 40-44, then 30–34 and 20-24, with the lowest still in 50–54 years. This indicates a shift over time, with the burden concentrating more towards women aged 35–44 years. Furthermore, prevalent case numbers were higher across all age groups in 2021 compared to 1990 (**Figure 5**).

### 3.4 The impact of SDI on the burden of PMS

**Figure 6:**
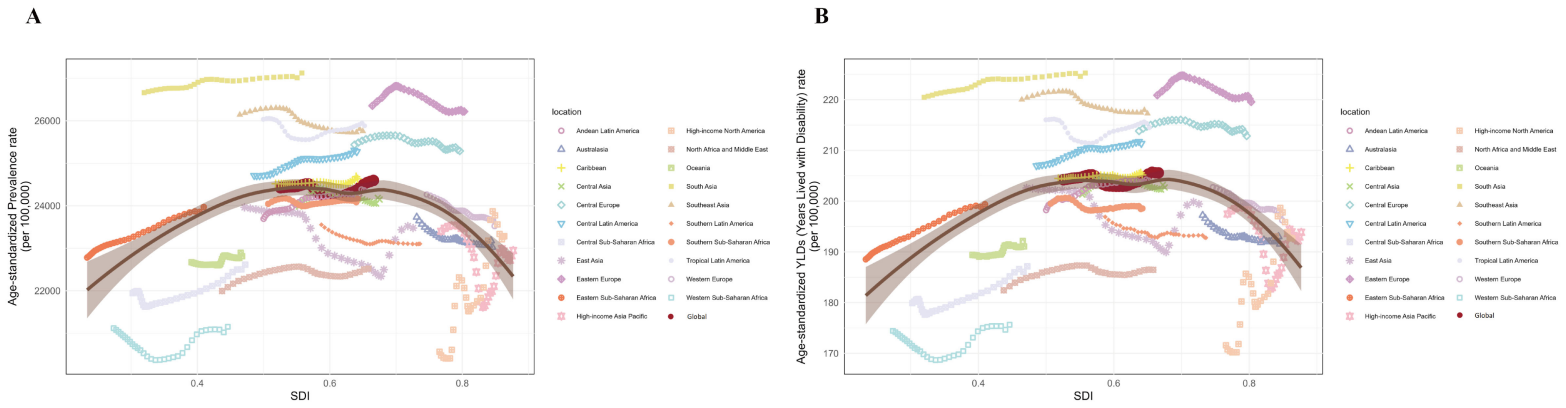
Non-linear relationships and trends between SDI with ASPR and age-standardized YLDs rate of premenstrual syndrome in 21 regions and globally from 1990 to 2021. **(A)** SDI with ASPR; **(B)** SDI with age-standardized YLDs rate. ASPR, age-standardized prevalence rate; YLDs, years lived with disability; SDI, Socio-demographic Index.

Globally, no significant correlation was found between ASPR and SDI (r = 0.0372, *p* = 0.304 > 0.05) or between age-standardized YLDs rate and SDI (r = 0.0670, *p* = 0.0637 > 0.05). However, LOESS regression revealed an inverse U-shaped relationship between SDI and both ASPR and age-standardized YLDs rate, with an inflection point around SDI = 0.6 (**Figure 6**). Stratified analysis confirmed this: for locations with SDI > 0.6, ASPR and age-standardized YLDs rate were significantly negatively correlated with SDI (r = -0.337, *p* = 9.20 × 10^−11^; r = -0.336, *p* = 1.076 × 10^−10^, respectively). Conversely, for locations with SDI ≤ 0.6, ASPR and age-standardized YLDs rate were significantly positively correlated with SDI (r = 0.322, *p* = 1.46×10^−11^; r = 0.330, *p* = 4.16×10^−^¹², respectively) (**Figure 6**).

**Figure 7:**
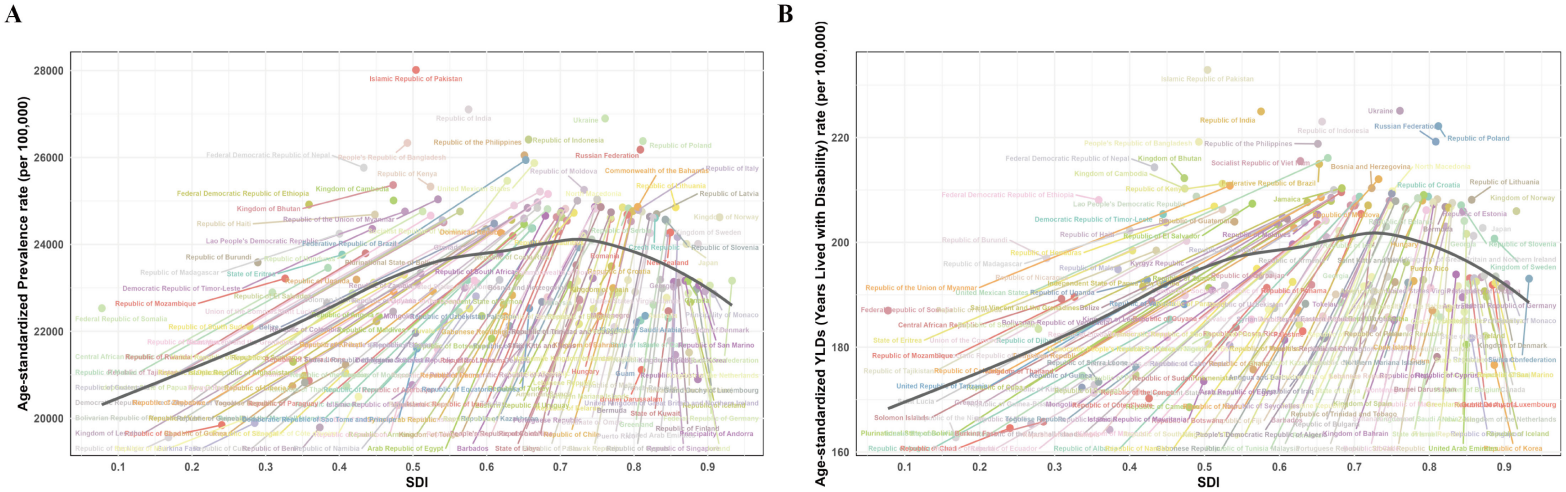
Non-linear relationship between SDI with ASPR and age-standardized YLDs rate of premenstrual syndrome in 204 countries and territories in 2021. **(A)** SDI with ASPR; **(B)** SDI with age-standardized YLDs rate. ASPR, age-standardized prevalence rate; YLDs, years lived with disability; SDI, Socio-demographic Index.

Correlation analysis across all 204 countries and territories showed a weak but significant positive correlation between SDI and ASPR (r = 0.172, *p* = 0.014) and between SDI and age-standardized YLDs rate (r = 0.206, *p* = 0.003). LOESS suggested an inflection point around SDI = 0.7 (**Figure 7**). Stratified analysis revealed: for locations with SDI > 0.7, ASPR and age-standardized YLDs rate were significantly negatively correlated with SDI (r = -0.283, *p* = 0.006; r = -0.291, *p* = 0.005, respectively). For locations with SDI ≤ 0.7, ASPR and age-standardized YLDs rate were significantly positively correlated with SDI (r = 0.427, *p* = 2.68×10^−^6; r = 0.434, *p* = 1.707×10^−^6, respectively) (**Figure 7**).

### 3.5 The projected burden of PMS from 2022 to 2050

**Figure 8:**
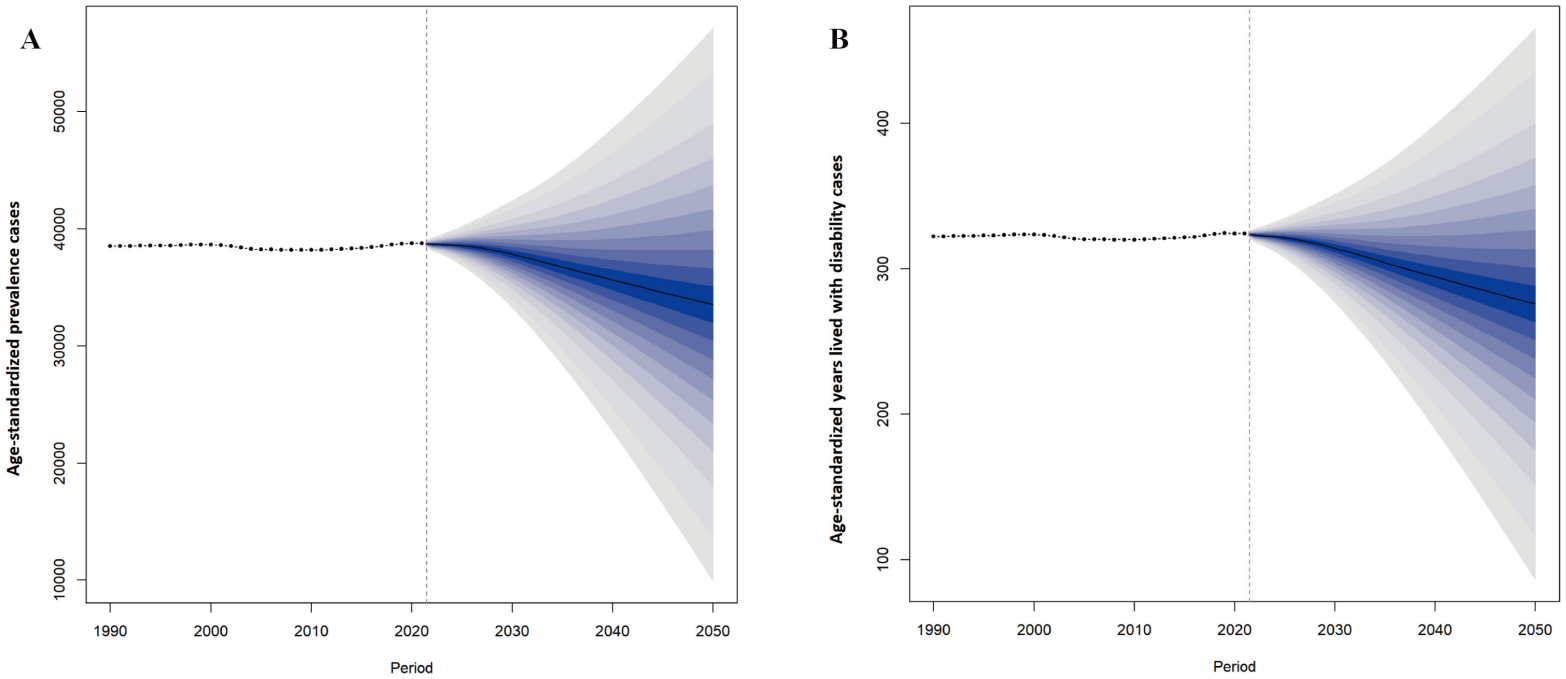
Projected changes in global premenstrual syndrome from 2022 to 2050. **(A)** ASPR; **(B)** Age-standardized YLDs rate. ASPR, age-standardized prevalence rate; YLDs, years lived with disability.

Projections based on the BAPC model showed a gradual decline in the global number of prevalence PMS cases by 2050. Similarly, projected global YLDs number for PMS also showed a decreasing trend (**Figure 8**). **Supplementary Table 3** provides detailed projections for prevalent cases and YLDs number globally from 2022 to 2050.

## 4 Discussion

PMS primarily manifesting as menstruation-related mood disorders, is associated with luteal-phase hormonal fluctuations, aberrant stress perception, and neurotransmitter imbalances (18, 19). The experience and reporting of emotions—particularly menstruation-related emotional symptoms—is strongly influenced by complex sociocultural and regional factors. Furthermore, the symptom-based diagnostic approach for PMS may contribute to underestimation of its true disease burden (14). Despite this, recent studies indicate PMS is a major driver of increased burden among combined gynecological diseases (20). Multiple studies demonstrate that females are disproportionately affected by mood disorders compared to males (21, 22), with depression historically exhibiting higher prevalence in female populations (23). This underscores significant gaps in attention to female menstrual mental health.

The United Nations Sustainable Development Goals explicitly recognized mental health and maternal health as central and intrinsic components of overall health (24). Global mental health has been inevitably impacted by complex healthcare, social, and public policy factors during the COVID-19 pandemic (25). Disparities in the allocation of macro-level societal resources are key determinants of global mental health inequalities. Promoting mental health equity through enhanced social services has thus emerged as a critical priority for transformative change (26, 27). Consequently, this study aims to provide insights for increasing awareness of female menstrual mental health, reducing regional inequalities, and advancing global mental health initiatives.

Utilizing GBD 2021 data, this study systematically analyzed dynamic trends in PMS burden globally, across five SDI regions, and in 204 countries/territories from 1990 to 2021, exploring age distribution patterns. We delved deeper into the correlation between SDI and PMS burden and employed the BAPC model to prospectively project burden trends up to 2050. Our results show that the global burden of PMS, measured by ASPR, ASIR, and age-standardized YLDs rate, increased in 2021 compared to 1990, including a U-shaped trend period (increase, decrease, then increase again) overall. Key risk factors for PMS include hormonal changes, stress, diet, and neurotransmitter imbalances, while medication use (including contraceptives), smoking, alcohol and caffeine consumption, and even education, age, and obstetric history also play roles (28). The rise in ASPR and ASIR is closely tied to population growth over the past three decades. Global socioeconomic progress has fostered greater awareness of mental health issues, including menstrual-related psychological problems, leading to increased recognition of PMS. Furthermore, evolving female societal roles, rising daily stress levels, increased negative emotions, and unhealthy lifestyles may contribute to the rising burden (29). The impact of the COVID-19 pandemic on women’s mental health likely also influenced PMS burden.

Among the five SDI regions, the low-middle SDI region consistently bore the heaviest burden, followed by the middle SDI region. The high SDI region generally had the lightest burden for most of 1990-2021. Notably, the low SDI region had the second-lightest burden after the high SDI region. Further correlation analyses at regional and national levels confirmed that ASPR and age-standardized YLDs rate exhibit an initial increase followed by a decrease with rising SDI. This non-linear relationship underscores a complex interplay between socioeconomic development and PMS burden (30). Initial socioeconomic improvements may coincide with increased stress among women and heightened awareness of menstrual mental health, yet constrained access to mental health services and medical care might increase diagnosis rates without adequate treatment, potentially worsening the burden. However, further socioeconomic advancement—bringing higher incomes, better education (potentially reducing stress), healthier lifestyles, more positive coping mechanisms, and improved healthcare access—likely contributes to burden reduction (14). Conversely, in low SDI settings, factors like inadequate healthcare, gender marginalization, menstrual stigma, limited PMS awareness, and restricted symptom expression likely lead to severe underdiagnosis (1, 14).

The age distribution of PMS prevalence shifted from a peak in the 20–24 age group in 1990 to the 35–39 group in 2021, followed by 40-44. This suggests a concentration of PMS prevalence among women aged 35–44 years over time. Women in this age group often face multiple stressors related to career, childcare, and eldercare, compounded by evolving societal roles, making them potentially more vulnerable to PMS. PMS itself can impair work efficiency, family relationships, and social interactions, potentially exacerbating symptoms and significantly impacting daily life (31). Stress is strongly linked to PMS; studies report higher PMS incidence among nurses under high stress (32). PMDD patients report experiencing acutely elevated daily stress (33), and PMS patients exhibit heightened stress sensitivity premenstrually (34). Stress is also a key factor in symptom exacerbation (35), possibly mediated by hypothalamic-pituitary-adrenal (HPA) and hypothalamic-pituitary-ovarian (HPO) axis interactions, central neurotransmitter imbalances, and neuroinflammation (18). Furthermore, PMS is a predictor of perinatal generalized anxiety (36). Therefore, greater societal attention and resources are needed for the mental health of reproductive-aged women, particularly those aged 35-44. Furthermore, the development of appropriate stress management and intervention measures, along with more comprehensive mental health support strategies, is considered a key focus for future research (37).

Fluctuations in ovarian hormone levels represent a core feature of PMS, which may be closely linked to the fact that ovarian steroids (estrogens, progestogens, and their metabolites) have receptor sites across multiple brain regions, thereby influencing neurotransmitter systems (8).Studies indicate that elevated levels of both estrogens and progestogens are involved in the occurrence of premenstrual depression, with a more pronounced association observed for progestogens (38).The underlying causes of these hormonal fluctuations include, on one hand, the interaction between the HPA and HPO axis triggered by stressors as previously discussed. On the other hand, dietary factors play a significant and non-negligible role. A cross-sectional analysis from China revealed that the traditional south China diet, characterized by high intake of rice and animal protein, was inversely associated with both PMS and PMDD, and this association was not explained by comorbid depression or anxiety symptoms (39). Additionally, dietary patterns high in calories, red and processed meats, fats, excessive caffeine, and sodium have been identified as risk factors for PMS-related symptoms (40, 41), Consistent evidence suggests potential benefits from adequate supplementation of vitamin B6 (≥50 mg/day), calcium (≥1000 mg/day), and zinc (≥30 mg/day) for PMS management (42).It is particularly noteworthy that while red meat and poultry are good sources of B vitamins, iron, and zinc—nutrients associated with reduced PMS risk—their origin from predominant artificial breeding practices increases the likelihood of containing high levels of exogenous hormones. Dietary intake of such products may elevate total and free estradiol levels while reducing sex hormone-binding globulin levels, ultimately exerting adverse effects on PMS (39).These findings highlight the importance of dietary modifications—such as reducing intake of red meat, poultry, high-calorie foods, caffeine, and sodium—along with appropriate nutrient supplementation (e.g., vitamin B6, calcium, zinc) for populations in high-burden regions and high-risk age groups for PMS.

Projections based on the BAPC model indicate declining trends in global age-standardized prevalent cases and YLDs for PMS by 2050, partly attributable to global population aging. However, given rising stress levels among women, PMS remains a significant concern. The substantial physical and mental burden PMS imposes throughout a woman’s life (4), and its association with personality disorders and suicide risk (19), demand attention. Current pharmacological treatments, including Selective Serotonin Reuptake Inhibitors, Combined Oral Contraceptives, and Gonadotropin-Releasing Hormone Agonists, are effective but have limitations due to side effects and potential impacts on fertility (2). The growing recognition of non-pharmacological therapies offers advantages. This suggests that closer collaboration between mental health services and medical systems at the societal level could be a practically significant step towards reducing the PMS burden and promoting global women’s mental health.

PMS is strongly influenced by social, economic, and cultural factors. Effective PMS management requires not only medical attention but also societal, cultural, and economic support. This integrated approach is essential to reduce regional disparities in PMS awareness and healthcare access, enabling early identification and intervention for high-risk and affected individuals through mental health services and medical care. To accurately analyze and reduce the global burden of PMS, equitable allocation of healthcare resources is paramount in resource-limited settings, focus should be prioritized on socioeconomically and medically underdeveloped regions. Enhancing menstrual health literacy and healthcare infrastructure in low SDI regions is critically important. Key measures include expanding basic healthcare infrastructure, implementing widespread community-based psychoeducational initiatives on menstrual health, increasing financial support for healthcare, enhancing the emphasis on women’s physiological and mental health curricula in educational institutions, and utilizing books, journals, websites, and other media to help eliminate stigma and raise public awareness of menstruation-related psychological health in low-development settings. Middle SDI regions require more robust mental health services and broader promotion of lifestyle management strategies, through coordinated efforts between medical institutions and community-based mental health services, scientifically grounded stress management and coping strategies can be implemented, thereby promoting comprehensive PMS improvement and safeguarding women’s menstrual-related mental well-being. Such efforts hold significant promise for mitigating the global burden of PMS and its societal impacts, ultimately enhancing global mental health. However, scientifically sound and efficient policy preference as well as specific resource allocation strategies for healthcare and social support still require in-depth research and exploration. Furthermore, individualized PMS management models, which encompassing medical intervention, mental health support, dietary adjustment, stress management, and physical activity. They need further refinement and broader dissemination.

## 5 Limitations

Although this study offers valuable insights into the global burden of PMS, several limitations should be acknowledged and addressed in future work (1): Due to database constraints, this study relied on GBD 2021 data for the global PMS burden analysis. In addition, the lack of PMS-specific risk factor data limited further analysis of their impact, potentially restricting the depth of discussion on preventive strategies. (2) PMS is significantly influenced by sociocultural factors, including stigma, limited awareness, and low health system coverage—particularly in low-SDI regions—leading to underreporting and nondisclosure, which may introduce bias and result in underestimation of the true PMS burden. (3) Predictive models cannot fully account for future policy adjustments across regions, which may lead to discrepancies between projected estimates and actual future burden.

## 6 Conclusion

The global burden of PMS has increased since 1990 and demonstrates an inverse U-shaped relationship with SDI. However, the true burden in low-SDI regions is likely severely underestimated due to substantial underreporting and nondisclosure. Regarding age, compared to 1990, the burden in 2021 shifted towards women aged 35-44. While focusing on women’s mental health across the lifespan, increased attention to the mental health, particular attention should be given to stress management of 35-44 age group. Furthermore, regions with underdeveloped socioeconomic and healthcare conditions require increased attention, including financial support, policy prioritization, emphasis on basic education, and assistance from media channels. In low-SDI regions, efforts should focus on raising public awareness of menstruation-related psychological issues and improving access to medical resources and psychological support. Meanwhile, low-middle and middle SDI regions should prioritize closer collaboration between community-based psychological services and healthcare systems, along with the development of effective stress management strategies. These measures will help enhance the equity of medical and psychological service resources, alleviate the global burden of PMS, and safeguard women’s mental health.

## Data Availability

The original contributions presented in the study are included in the article/**Supplementary Material**. Further inquiries can be directed to the corresponding author.

## References

[1] Howard LM Wilson CA Reilly TJ Moss KM Mishra GD Coupland-Smith E . Women's reproductive mental health: currently available evidence and future directions for research, clinical practice and health policy. World Psychiatry. (2025) 24:196–215. doi: 10.1002/wps.21305, PMID: 40371748 PMC12079463

[2] Committee on Clinical Practice Guidelines–Gynecology . Management of premenstrual disorders: ACOG clinical practice guideline no. 7. Obstet Gynecol. (2023) 142:1516–33. doi: 10.1097/aog.0000000000005426, PMID: 37973069

[3] Epperson CN Steiner M Hartlage SA Eriksson E Schmidt PJ Jones I . Premenstrual dysphoric disorder: evidence for a new category for DSM-5. Am J Psychiatry. (2012) 169:465–75. doi: 10.1176/appi.ajp.2012.11081302, PMID: 22764360 PMC3462360

[4] Standeven LR Bajaj M McEvoy K Shirinian D Voegtline K Osborne LM . The link between childhood traumatic events and the continuum of premenstrual disorders. Front Psychiatry. (2024) 15:1443352. doi: 10.3389/fpsyt.2024.1443352, PMID: 39444627 PMC11496889

[5] Yang Y Valdimarsdóttir UA Manson JE Sievert LL Harlow BL Eliassen AH . Premenstrual disorders, timing of menopause, and severity of vasomotor symptoms. JAMA Netw Open. (2023) 6:e2334545. doi: 10.1001/jamanetworkopen.2023.34545, PMID: 37725375 PMC10509727

[6] Hantsoo L Rangaswamy S Voegtline K Salimgaraev R Zhaunova L Payne JL . Premenstrual symptoms across the lifespan in an international sample: data from a mobile application. Arch Womens Ment Health. (2022) 25:903–10. doi: 10.1007/s00737-022-01261-5, PMID: 36018464 PMC9492621

[7] Reilly TJ Patel S Unachukwu IC Knox CL Wilson CA Craig MC . The prevalence of premenstrual dysphoric disorder: Systematic review and meta-analysis. J Affect Disord. (2024) 349:534–40. doi: 10.1016/j.jad.2024.01.066, PMID: 38199397

[8] Henderson A Gardani M Dyker G Matthews L . Cognition and behaviour across the menstrual cycle in individuals with premenstrual dysphoric disorder - A systematic review. J Affect Disord. (2025) 371:134–46. doi: 10.1016/j.jad.2024.11.033, PMID: 39577497

[9] Ohseto H Takahashi I Narita A Obara T Ishikuro M Kobayashi N . Risk factors, prognosis, influence on the offspring, and genetic architecture of perinatal depression classified based on the depressive symptom trajectory. Depress Anxiety. (2024) 2024:6622666. doi: 10.1155/2024/6622666, PMID: 40226751 PMC11918876

[10] Eisenlohr-Moul T Divine M Schmalenberger K Murphy L Buchert B Wagner-Schuman M . Prevalence of lifetime self-injurious thoughts and behaviors in a global sample of 599 patients reporting prospectively confirmed diagnosis with premenstrual dysphoric disorder. BMC Psychiatry. (2022) 22:199. doi: 10.1186/s12888-022-03851-0, PMID: 35303811 PMC8933886

[11] Maqbool R Maqbool M Zehravi M Ara I . Menstrual distress in females of reproductive age: a literature review. Int J Adolesc Med Health. (2021) 34:11–7. doi: 10.1515/ijamh-2021-0081, PMID: 34293834

[12] Murray CJL . The Global Burden of Disease Study at 30 years. Nat Med. (2022) 28:2019–26. doi: 10.1038/s41591-022-01990-1, PMID: 36216939

[13] GBD 2021 Diseases and Injuries Collaborators . Global incidence, prevalence, years lived with disability (YLDs), disability-adjusted life-years (DALYs), and healthy life expectancy (HALE) for 371 diseases and injuries in 204 countries and territories and 811 subnational locations, 1990-2021: a systematic analysis for the Global Burden of Disease Study 2021. Lancet. (2024) 403:2133–61. doi: 10.1016/s0140-6736(24)00757-8, PMID: 38642570 PMC11122111

[14] Liu X Li R Wang S Zhang J . Global, regional, and national burden of premenstrual syndrome, 1990-2019: an analysis based on the Global Burden of Disease Study 2019. Hum Reprod. (2024) 39:1303–15. doi: 10.1093/humrep/deae081, PMID: 38689567

[15] GBD 2021 Risk Factors Collaborators . Global burden and strength of evidence for 88 risk factors in 204 countries and 811 subnational locations, 1990-2021: a systematic analysis for the Global Burden of Disease Study 2021. Lancet. (2024) 403:2162–203. doi: 10.1016/s0140-6736(24)00933-4, PMID: 38762324 PMC11120204

[16] Dongjun Z Mingyue W Xinqi L Lina W Jiali W Mengyao J . Trends in depressive and anxiety disorders among adolescents and young adults (aged 10-24) from 1990 to 2021: A global burden of disease study analysis. J Affect Disord. (2025) 387:119491. doi: 10.1016/j.jad.2025.119491, PMID: 40441639

[17] Xu M Wei H Lv D Wei Y Liu Z Zhang Y . Trends and future predictions of chronic kidney disease due to diabetes mellitus type 2 attributable to dietary risks: insights based on GBD 2021 data. Front Nutr. (2024) 11:1494383. doi: 10.3389/fnut.2024.1494383, PMID: 39872139 PMC11769828

[18] Cheng M Jiang Z Yang J Sun X Song N Du C . The role of the neuroinflammation and stressors in premenstrual syndrome/premenstrual dysphoric disorder: a review. Front Endocrinol (Lausanne). (2025) 16:1561848. doi: 10.3389/fendo.2025.1561848, PMID: 40225329 PMC11985436

[19] Kulkarni J Mu E . Premenstrual exacerbation of psychiatric symptoms: from misdiagnosis to management. Br J Psychiatry. (2025) 226:346–8. doi: 10.1192/bjp.2024.295, PMID: 40468821

[20] Gao Y Wang X Wang Q Jiang L Wu C Guo Y . Rising global burden of common gynecological diseases in women of childbearing age from 1990 to 2021: an update from the Global Burden of Disease Study 2021. Reprod Health. (2025) 22:57. doi: 10.1186/s12978-025-02013-1, PMID: 40259342 PMC12010537

[21] Boyd A Van de Velde S Vilagut G de Graaf R O'Neill S Florescu S . Gender differences in mental disorders and suicidality in Europe: results from a large cross-sectional population-based study. J Affect Disord. (2015) 173:245–54. doi: 10.1016/j.jad.2014.11.002, PMID: 25462424

[22] Fan Y Fan A Yang Z Fan D . Global burden of mental disorders in 204 countries and territories, 1990-2021: results from the global burden of disease study 2021. BMC Psychiatry. (2025) 25:486. doi: 10.1186/s12888-025-06932-y, PMID: 40375174 PMC12080068

[23] Herrman H Patel V Kieling C Berk M Buchweitz C Cuijpers P . Time for united action on depression: a Lancet-World Psychiatric Association Commission. Lancet. (2022) 399:957–1022. doi: 10.1016/s0140-6736(21)02141-3, PMID: 35180424

[24] Patel V Saxena S Lund C Thornicroft G Baingana F Bolton P . The Lancet Commission on global mental health and sustainable development. Lancet. (2018) 392:1553–98. doi: 10.1016/s0140-6736(18)31612-x, PMID: 30314863

[25] Aknin LB Andretti B Goldszmidt R Helliwell JF Petherick A De Neve JE . Policy stringency and mental health during the COVID-19 pandemic: a longitudinal analysis of data from 15 countries. Lancet Public Health. (2022) 7:e417–26. doi: 10.1016/s2468-2667(22)00060-3, PMID: 35461592 PMC9023007

[26] Burgess RA Jain S Petersen I Lund C . Social interventions: a new era for global mental health? Lancet Psychiatry. (2020) 7:118–9. doi: 10.1016/s2215-0366(19)30397-9, PMID: 31653556

[27] Larrieta J Wuerth M Aoun M Bemme D D'Souza N Gumbonzvanda N . Equitable and sustainable funding for community-based organisations in global mental health. Lancet Glob Health. (2023) 11:e327–8. doi: 10.1016/s2214-109x(23)00015-3, PMID: 36716753

[28] Modzelewski S Oracz A Żukow X Iłendo K Śledzikowka Z Waszkiewicz N . Premenstrual syndrome: new insights into etiology and review of treatment methods. Front Psychiatry. (2024) 15:1363875. doi: 10.3389/fpsyt.2024.1363875, PMID: 38716118 PMC11075635

[29] Zolfaghary F Adib-Rad H Nasiri-Amiri F Faramarzi M Pasha H Gholinia-Ahangar H . Effectiveness of computer-based stress inoculation training (SIT) counseling approach on anxiety, depression, and stress of students with premenstrual syndrome. BMC Public Health. (2024) 24:555. doi: 10.1186/s12889-024-18003-0, PMID: 38388370 PMC10882748

[30] Tan J Shu Y Li Q Liang L Zhang Y Zhang J . Global, regional, and national burden of self-harm among adolescents aged 10–24 years from 1990 to 2021, temporal trends, health inequities and projection to 2041. Front Psychiatry. (2025) 16:1564537. doi: 10.3389/fpsyt.2025.1564537, PMID: 40225845 PMC11986636

[31] Brown D Smith DM Osborn E Wittkowski A . The experiences and psychological impact of living with premenstrual disorders: a systematic review and thematic synthesis. Front Psychiatry. (2024) 15:1440690. doi: 10.3389/fpsyt.2024.1440690, PMID: 39286397 PMC11402655

[32] Wang X Ge Y Liu Y Hu W Wang Y Yu S . The association between occupational stress, sleep quality and premenstrual syndrome among clinical nurses. BMC Nurs. (2024) 23:661. doi: 10.1186/s12912-024-02329-6, PMID: 39289708 PMC11409569

[33] Beddig T Reinhard I Kuehner C . Stress, mood, and cortisol during daily life in women with Premenstrual Dysphoric Disorder (PMDD). Psychoneuroendocrinology. (2019) 109:104372. doi: 10.1016/j.psyneuen.2019.104372, PMID: 31357135

[34] Gonda X Fountoulakis KN Csukly G Telek T Pap D Rihmer Z . Association of a trait-like bias towards the perception of negative subjective life events with risk of developing premenstrual symptoms. Prog Neuropsychopharmacol Biol Psychiatry. (2010) 34:500–5. doi: 10.1016/j.pnpbp.2010.02.004, PMID: 20138198

[35] Suzuki YC Ohira H . Women with premenstrual syndrome exhibit high interoceptive accuracy, but low awareness, with parasympathetic rebound responses from stress. Front Neurosci. (2025) 19:1489225. doi: 10.3389/fnins.2025.1489225, PMID: 40035061 PMC11872929

[36] Ventriglio A Severo M Petito A Nappi L Iuso S Altamura M . Associated factors to Generalized Anxiety in a sample of women screened for the risk of perinatal depression: a naturalistic study from Italy. J Affect Disord. (2025) 386:119474. doi: 10.1016/j.jad.2025.119474, PMID: 40419144

[37] Yang Y Jia Y Fu S Zhang L Xiang F Hu W . The prevalence of premenstrual syndrome in China: a systematic review and meta-analyses. Front Psychiatry. (2025) 16:1640781. doi: 10.3389/fpsyt.2025.1640781, PMID: 41019595 PMC12461857

[38] WeiSM WakimP MartinezPE NiemanLK RubinowDR SchmidtPJ . Differential effects of ovarian steroids in women with and without premenstrual dysphoric disorder: A replication and extension of findings. Am J Psychiatry. (2025) 182:922–34. doi: 10.1176/appi.ajp.20240596, PMID: 41030005

[39] Shi X Chen M Pan Q Zhou J Liu Y Jiang T . Association between dietary patterns and premenstrual disorders: a cross-sectional analysis of 1382 college students in China. Food Funct. (2024) 15:4170–9. doi: 10.1039/d3fo05782h, PMID: 38482855

[40] Mighani S Shivyari FT Razzaghi A Amerzadeh M Javadi M . Relationship between dietary intake, eating attitudes, and premenstrual syndrome severity among Iranian women: insights from a cross-sectional study. J Eat Disord. (2025) 13:131. doi: 10.1186/s40337-025-01326-7, PMID: 40640956 PMC12243156

[41] Oboza P Ogarek N Wójtowicz M Rhaiem TB Olszanecka-Glinianowicz M Kocełak P . Relationships between premenstrual syndrome (PMS) and diet composition, dietary patterns and eating behaviors. Nutrients. (2024) 16:1911. doi: 10.3390/nu16121911, PMID: 38931266 PMC11206370

[42] Robinson J Ferreira A Iacovou M Kellow NJ . Effect of nutritional interventions on the psychological symptoms of premenstrual syndrome in women of reproductive age: a systematic review of randomized controlled trials. Nutr Rev. (2025) 83:280–306. doi: 10.1093/nutrit/nuae043, PMID: 38684926 PMC11723155

